# Supplementary figures and images for: An integrative pan-cancer investigation reveals common genetic and transcriptional alterations of AMPK pathway genes as important predictors of clinical outcomes across major cancer types
Source: BMC Cancer. 2020 Aug 17;20:773. doi: 10.1186/s12885-020-07286-2 (PMC7433212; doi:10.1186/s12885-020-07286-2)

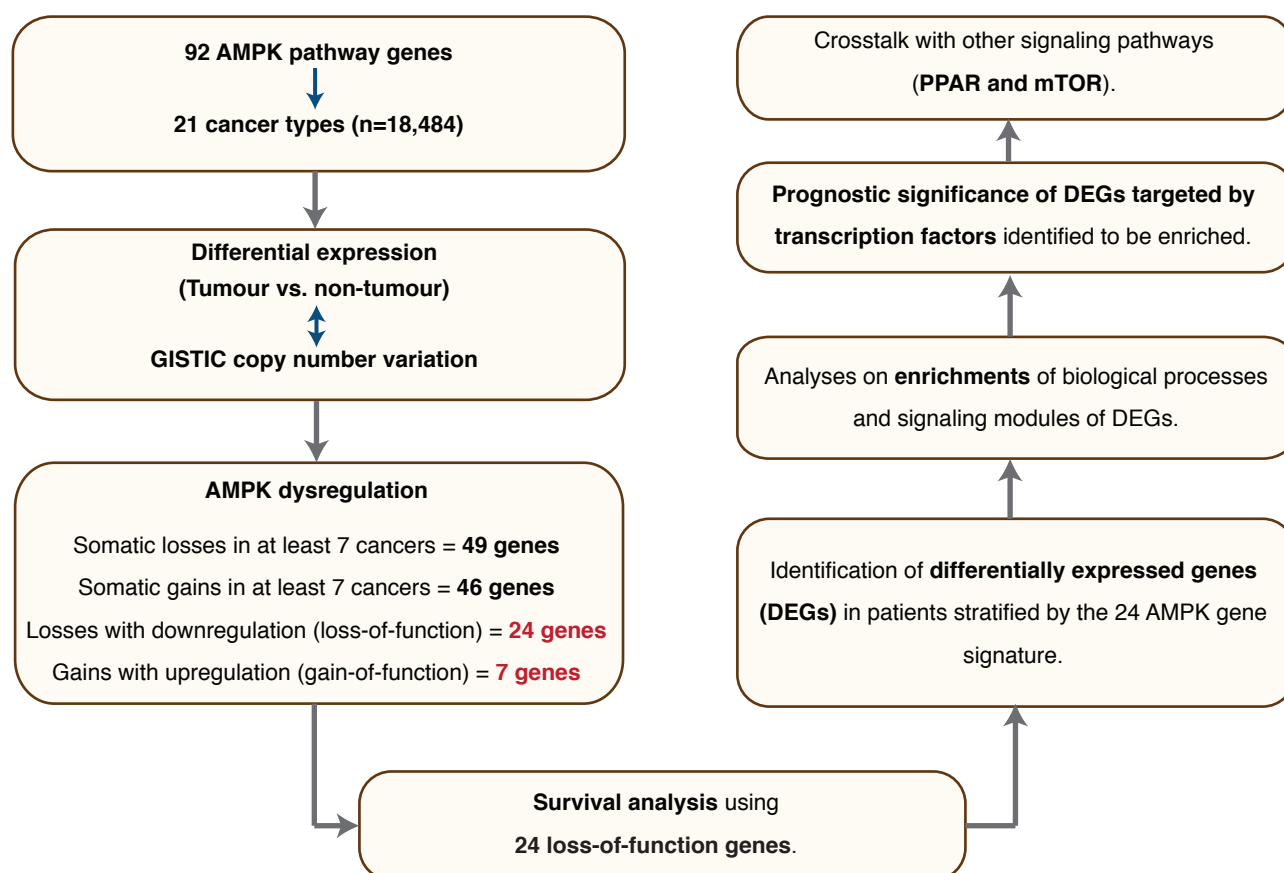

Supplement: Supplementary file 2 — Additional file 2. Flowchart depicting the study design. [file 12885_2020_7286_MOESM2_ESM.pdf]

A

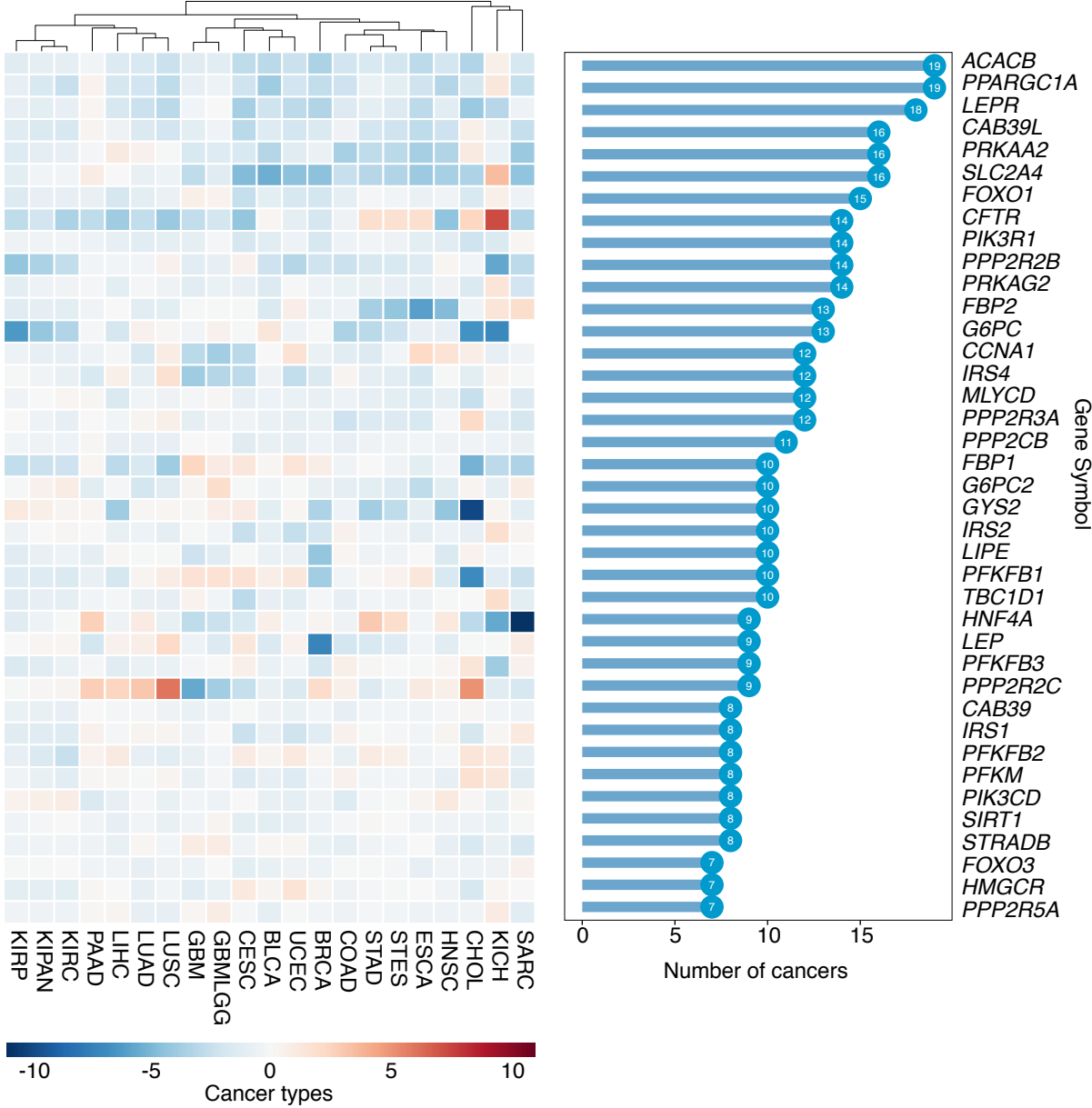

B

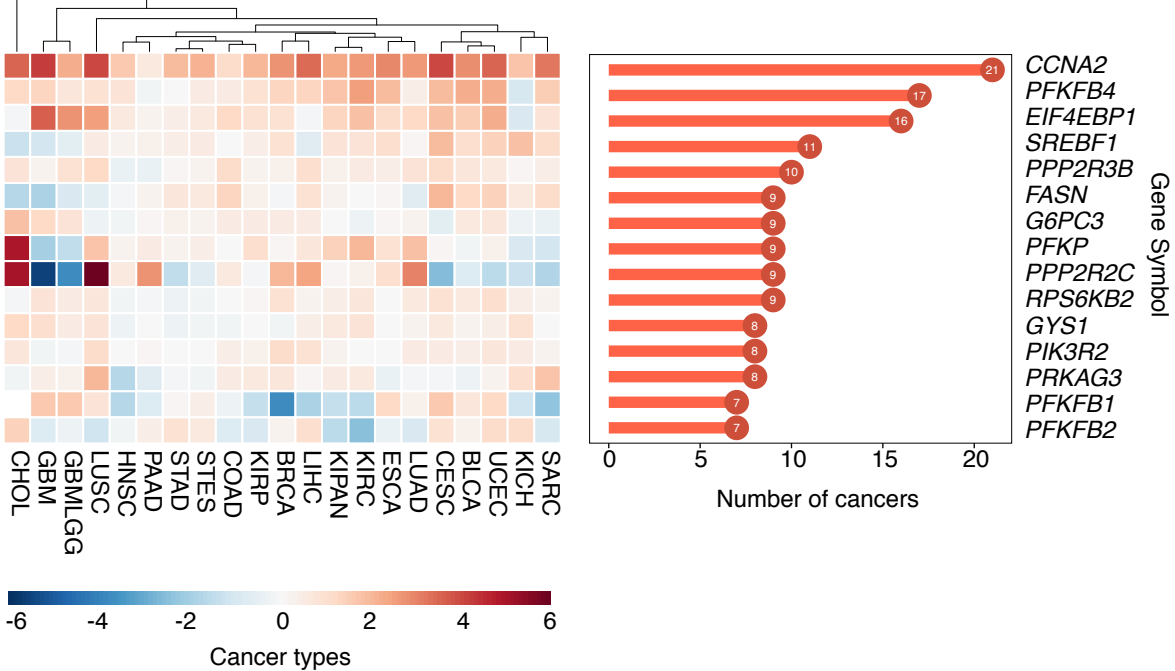

Supplement: Supplementary file 4 — Additional file 4 Differential transcript expression of AMPK pathway genes. Differential expression analyses performed on tumor vs. non-tumor samples reveal (A) 39 significantly downregulated genes and (B) 15 significantly upregulated genes. Only genes that are differentially expressed in at least seven cancer types are shown. The bar charts on the right of each heatmap depict the number of cancers affected by altered transcript expression. [file 12885_2020_7286_MOESM4_ESM.pdf]
